# Contacts with primary and secondary healthcare prior to suicide: case–control whole-population-based study using person-level linked routine data in Wales, UK, 2000–2017

**DOI:** 10.1192/bjp.2020.137

**Published:** 2020-12

**Authors:** Ann John, Marcos DelPozo-Banos, David Gunnell, Michael Dennis, Jonathan Scourfield, David V. Ford, Nav Kapur, Keith Lloyd

**Affiliations:** 1 Department of Population Psychiatry, Suicide and Informatics, Swansea University Medical School; and Public Health Wales NHS Trust, UK; 2 Department of Medicine, Swansea University Medical School, UK; 3 Department of Population Health Sciences, Bristol Medical School; and NIHR Biomedical Research Centre at University Hospitals Bristol and Weston NHS Foundation Trust, UK; 4 Swansea University Medical School, UK; 5 School of Social Sciences, Cardiff University, UK; 6 Division of Psychology and Mental Health, University of Manchester; and Greater Manchester Mental Health NHS Foundation Trust; and NIHR Greater Manchester Patient Safety Translational Research Centre, UK

**Keywords:** Suicide, self-harm, electronic health records, primary care, emergency department

## Abstract

**Background:**

Longitudinal studies of patterns of healthcare contacts in those who die by suicide to identify those at risk are scarce.

**Aims:**

To examine type and timing of healthcare contacts in those who die by suicide.

**Method:**

A population-based electronic case–control study of all who died by suicide in Wales, 2001–2017, linking individuals’ electronic healthcare records from general practices, emergency departments and hospitals. We used conditional logistic regression to calculate odds ratios, adjusted for deprivation. We performed a retrospective continuous longitudinal analysis comparing cases’ and controls’ contacts with health services.

**Results:**

We matched 5130 cases with 25 650 controls (5 per case). A representative cohort of 1721 cases (8605 controls) were eligible for the fully linked analysis. In the week before their death, 31.4% of cases and 15.6% of controls contacted health services. The last point of contact was most commonly associated with mental health and most often occurred in general practices. In the month before their death, 16.6 and 13.0% of cases had an emergency department contact and a hospital admission respectively, compared with 5.5 and 4.2% of controls. At any week in the year before their death, cases were more likely to contact healthcare services than controls. Self-harm, mental health and substance misuse contacts were strongly linked with suicide risk, more so when they occurred in emergency departments or as emergency admissions.

**Conclusions:**

Help-seeking occurs in those at risk of suicide and escalates in the weeks before their death. There is an opportunity to identify and intervene through these contacts.

Suicide and suicidal behaviours are recognised as important issues for public health policy and practice in the UK and globally. Although there has been some progress in reducing suicide mortality globally, much of the decline is driven by decreases in China and India^1^ and this is more likely attributable to improved living standards than specific suicide prevention efforts. A recent meta-analysis identified a general lack of progress in the suicide prevention field in the past 50 years.^2^ The authors highlight the scarcity of studies on short-term or acute risk and of longitudinal studies of proximal warning signs for suicidal behaviour. Many people who die by suicide have been in contact with health services in the year before their death.^3–15^ This provides a unique opportunity for intervention.

Previous studies have focused on suicide following discharge from psychiatric hospitals, with long-standing consistent findings of a peak incidence of deaths occurring within 3 months of discharge.^3^ A few studies have extended their analyses to all those who have died by suicide (i.e. known and unknown to mental health services) and measured the proportion of those in contact with health services prior to their death.^4,5^ They found that fewer than 1 in 3 of those who die by suicide are in contact with mental health services in the year before they die and that the majority (49–92%) make contact with primary healthcare.^4,5^ Most suicide studies combining primary and secondary care data do not include population-representative controls for comparison.^6^ The ones that do are small in scale and/or based on data collected by interviewing next of kin and healthcare professionals.^7^ The only large-scale (over 4000 deaths by suicide) case–control population-based study analysed primary care data only.^8^ There is a need for suicide studies with population-representative controls examining the type and cause of contacts across all health services.

To the best of our knowledge, ours is the first study on suicide combining all of the following characteristics: a population-based data-set; a study period of 17 years; linkage of administrative, primary, emergency department and secondary care data at a person level; a case–control design with live controls (at the time of the cases’ death) drawn from the general population; and a continuous longitudinal analysis over the last 12 months of life. We aim to explore the type and time of healthcare services contacted by those who die by suicide in the year before their death in order to identify potential opportunities for prevention.

## Method

### Study design and participants

Wales, a country within the UK, has a population of 3.1 million. Of the approximately 33 000 deaths registered each year, around 350 are suicides or events of undetermined intent.^9^ It is conventional practice to include the latter in the definition of suicide.

The Suicide Information Database-Wales (SID-Cymru) is a population-based electronic cohort of individuals who died by suicide in Wales (cases) and matched controls hosted in the Secure Anonymised Information Linkage (SAIL) Databank.^10^ The SAIL Databank (www.saildatabank.com) is an expanding resource of anonymised secure privacy-protecting person-based linkable data from health and public settings to support research. SAIL assigns an anonymous linkage field unique for each individual using a privacy-protecting split file approach, which can then be used to link across data-sets at a person level. We used all records linked deterministically or probabilistically with a matching score ≥0.9.^11^

SID-Cymru defines those who died by suicide (cases) using ICD-10 codes for suicide (X60–84) and undetermined deaths (Y10-34, Y87, and Y87.2; excluding Y33.9 before 2007) recorded in the Office for National Statistics deaths register.^10^ The minimum age at death for inclusion was 10 years for suicide and 15 years for undetermined deaths.^9^ Our study population includes deaths between 1 January 2001 and 31 December 2017. We set the index date for each case as the date of death or the start date of a healthcare contact during which their death occurred (e.g. following an emergency admission due to overdose or during a psychiatric admission). The contact prior to the index date is where an opportunity for prevention exists.

Controls were selected using incidence density sampling. We randomly matched each case to five controls by gender and week of birth (±1 year).^10^ Controls were alive at the time of the case's death, to minimise bias,^12^ and were unique. During control selection, those with a similar period of Welsh residency were chosen to ensure similar quality of data coverage.

Six data-sets were utilised in our analysis: the Welsh Demographic Service; Office for National Statistics – Deaths; the General Practice Database (GPD – covering 77%, i.e. 333/432, of all general practices in Wales); Emergency Department Data Set for NHS Wales; Patient Episode Database for Wales; and the Outpatient Data Set for NHS Wales (full details are in supplementary Table 1, available at https://doi.org/10.1192/bjp.2020.137). Owing to variable data availability across individual data-sets, we used: (a) those registered with a general practice providing data to SAIL in the year before their index date to study general practice contacts; (b) those with an index date in 2010 or later to study emergency department contacts; (c) the whole-Wales population to study hospital admissions; (d) those with an index date in 2005 or later to study hospital out-patient contacts; and (e) all those with an index date in 2010 or later and registered with a general practice providing data to SAIL in the year before their index date to simultaneously study contacts across all four settings.

SAIL's Information Governance Review Panel granted ethical approval to conduct this research (IGRP number 0204). Under permissions granted to the SAIL Databank, individuals' informed consent was not necessary and all data was anonymised.

### Measures

Four demographic variables were extracted at the point of the index date from the Welsh Demographic Service data-set: gender; age categories (10–24, 25–64, >65 years); level of deprivation; and rural/urban context. Variables dependent on geographical location were extracted from general practice residential records in the GPD. We used quintiles of the Welsh Index of Multiple Deprivation (WIMD) 2011 score as a measure of area deprivation at lower-layer super-output area level; approximately 1500 individuals per area level.^13^ WIMD deprivation levels 1 to 5 were defined using national WIMD score quintiles as cut-offs, with level 1 representing the lowest deprivation areas. Rural/urban context is an indicator for England and Wales categorising each area into ‘urban’ (i.e. settlement types with a population of 10 000 or more) and ‘rural’ (i.e. the union of the categories ‘town and fringe’ and ‘village, hamlet and isolated dwellings’).^14^

We examined all general practice, emergency department, and hospital in-patients and out-patients contacts for each case/control before their index date in each setting's data-set separately. We defined ‘contact’ as a recorded entry in the GPD, Emergency Department Data Set, Patient Episode Database for Wales or Outpatient Data Set. In the GPD, we excluded administrative codes and associated diagnoses such as ‘letter from emergency department’, but did include telephone and face-to-face contacts with any member of the general practice team. We also identified specific types of contact in the year leading to the index date across data-sets.

We identified specific types of contact (e.g. mental health, substance misuse diagnoses, prescriptions) in the year leading to the index date across data-sets (supplementary Table 2). Contacts were classified on the basis of validated read-code and ICD-10 code lists and with the input of expert clinicians (supplementary Table 3, including references). We stratified hospital admissions into emergency and elective admissions. Any contact may be associated with more than one type of diagnostic or treatment category.

### Statistical analysis

We used SQL Db2 (www.ibm.com/analytics/db2) to interrogate data in the SAIL Databank and performed the analysis using R (www.r-project.org) for Windows. Descriptive statistics were used to summarise group characteristics and proportions of people and presenting complaints to each setting. This included counts, percentages and 95% confidence intervals (CIs) estimated by Wilson score with continuity correction.^15^ We used conditional logistic regression to compare ratios of cases and controls while adjusting for deprivation (WIMD quintile).

We measured contacts with health services using the linked data-set across all settings (general practice, emergency department, and hospital in-patients and out-patients) within 1 week, 1 month and 1 year before the index date. We recorded where the last contact in the year leading to the index date occurred and why. We examined whether a patient had a mental health or self-harm contact in any of the health data-sets within 1 year of the index date. We studied the trajectory of healthcare contacts before the index date, measuring: (a) the proportion of cases and controls with contacts in the 1 to 365 days before the index date and (b) the rate of cases and controls with contacts in a 1-week window starting at day 7 to day 365 before the index date. For the trajectory analysis only, we corrected *P*-values from each graph for multiple testing using false discovery rate.^16^

Finally, we conducted a sensitivity analysis repeating all analyses using only those with a coroner's conclusion of ‘suicide’, i.e. death by intentional self-harm, to ascertain any bias introduced by the inclusion of undetermined deaths.

## Results

We identified 5237 individuals who died by suicide in Wales within the study period, of whom 5130 (98%) had valid linkage and data quality and were used as the study population. Of these, 3999/5130 (78.0%) were male. At the time of death, 571/5130 (11.2%) were under 25 years old and 817/5130 (15.9%) were above 64. We matched these individuals with 25 650 controls (five per case). Of those who died by suicide, 2339/5130 (45.6%) lived in WIMD quintiles 4 and 5 areas, compared with 10 086/25 650 (39.3%) controls (for quintile 4, OR = 1.1 (95% CI 1.0–1.1); for quintile 5, OR = 1.3 (95% CI 1.3–1.4)). Of the cases, 3241/5130 (63.2%) resided in urban areas and 1576/5130 (30.7%) in rural areas, compared with 17 532/25 650 (68.3%) and 8084/25 650 (31.5%) respectively for the controls (OR = 0.7 (95% CI 0.6–0.7) for urban; and OR = 1.0 (95% CI 0.9–1.1) for rural). Of those who died by suicide, 557/5130 (10.9%) had a contact with services during which their death by suicide was recorded, and 397/5130 (7.8%) had such contact spanning 2 or more days (average length: 23.6 days; range: 1 day to 7.3 years. During this contact, 105/557 (18.9%) were under psychiatric care, 216/557 (38.8%) had a recorded mental health diagnosis and 258/557 (46.3%) a self-harm diagnosis.

Owing to variable data availability across individual data-sets, in the year leading to the index date, 3504/5130 cases (68.3%) had GPD data available, 2476/5130 cases (48.3%) had emergency department data, all 5130 cases had hospital in-patient data, 3861/5130 cases (75.3%) had hospital out-patients data, and 1721/5130 cases (33.5%) had data coverage across all settings (general practice, emergency department, hospital in-patients and hospital out-patients) (supplementary Fig. 1). All subpopulations of cases had similar demographic profiles to the full study population of cases, so it could be reasonably assumed that these organisational and administrative differences did not affect representativeness (supplementary Table 4).

### Type of contact

#### General practice

Most cases and controls had a general practice contact in the year leading to the index date. Table 1 shows full results for cases and controls with different types of general practice contact, with gender stratification in supplementary Table 5. All the studied types of contact were more likely in cases than in controls. The OR was distinctively highest for self-harm: OR = 33.1 (95% CI 23.8–45.9). All the studied contacts were more common in females than males for both cases and controls, with ORs relatively similar between them. The only notable exceptions, with ORs for females higher than males, were alcohol misuse contacts (OR = 225.7 (95% CI 31.6–1609.1) *v*. OR = 7.7 (95% CI 6.2–9.7)) and prescription of psychotropics (OR = 9.5 (95% CI 8.3–10.8) *v*. OR = 7.7 (95% CI 7.1–8.3)).

**Table 1:** Type of general practice contacts in the year before index date^a^

	Cases (*N* = 3504)		Controls (*N* = 17 520)		OR	95% CI
	*n* (%)	95% CI	*n* (%)	95% CI		
Any contact	2933 (83.8)	82.5–84.9	12 258 (70.0)	63.9–70.7	2.3	2.2–2.5
Recorded mental health specialty contact	359 (10.3)	9.3–11.3	161 (1)	0.8–1.1	12.4	10.5–14.8
Diagnoses						
Any diagnosis	2103 (60.1)	58.4–61.7	8141 (46.5)	45.8–47.3	1.8	1.7–1.9
Non-mental health	1892 (54.0)	52.4–55.7	7911 (45.2)	44.5–45.9	1.5	1.4–1.5
Mental health	1007 (28.8)	27.3–30.3	977 (5.6)	5.3–6.0	6.9	6.3–7.5
Common mental disorder	767 (21.9)	20.6–23.3	662 (3.8)	3.6–4.1	7.2	6.6–7.9
Injury and poisoning	228 (6.6)	5.8–7.4	752 (4.3)	4.1–4.7	1.6	1.4–1.7
Accidents	83 (2.4)	2.0–3.0	249 (1.5)	1.3–1.7	1.7	1.4–1.9
Accidental hanging and poisoning	<10 (0.3)	0.2–0.6	<5 (0.1)	0.1–0.1	4.3	0.9–19.8^b^
Self-harm	252 (7.2)	6.4–8.1	38 (0.3	0.2–0.3	33.1	23.8–45.9
Alcohol misuse	194 5.6	4.9–6.4	<99 (0.6)	0.5–0.7	10.2	8.2–12.6
Drugs misuse	87 (2.5)	2.1–3.1	69 (0.4)	0.4–0.5	6.1	4.7–7.8
Prescriptions						
Any prescription	2812 (80.3)	79.0–81.6	11 534 (65.9)	65.2–66.6	2.2	2.1–2.4
Opiates	918 (26.2)	24.8–27.7	2833 (16.2)	15.7–16.8	1.8	1.7–1.9
Psychotropics	1971 (56.3)	54.7–57.9	2574 (14.7)	14.2–15.3	8.1	7.6–8.6

a. Results from those with General Practice Database data in the year before index date. Odds ratios (OR) adjusted for deprivation. *P* < 0.001 for all results, unless otherwise shown.

b. *P* = 0.059.

#### Emergency department

In the year before their index date, 1008/5130 cases (40.8%) and 2028/12 380 controls (16.4%) had an emergency department attendance (OR = 3.5; 95% CI 3.3–3.7). ‘Injury and poisoning’ was the most common reason for attendance in both groups, with this arising in 422/5130 (17.1%) cases and 774/12 380 (6.3%) controls (OR = 3.0; 95% CI 2.8–3.3). This was followed by accidents, in 401/5130 (16.2%) cases and 984/12 380 (8%) controls (OR = 2.2; 95% CI 2.0–2.4). The remaining specific studied types of contact were very rare for controls (<0.2%) and rare for cases (<8%), resulting in large ORs and wide confidence intervals, e.g. for self-harm, OR = 38.7 (95% CI 25.6–58.6). Comparatively more females (255/521 (49%); OR = 5.1 (95% CI 4.4–5.9)) than males (753/1955 (38.6%); OR = 3.1 (95% CI 2.9–3.4)) had contact with an emergency department, a pattern observed across all studied types of emergency department contacts.

#### Secondary care

One-third of cases had a hospital admission in this period. Table 2 shows full results for cases and controls with different types of secondary care contact, with gender stratification in supplementary Table 6. Hospital admissions with a recorded mental health specialist contact, as well as both emergency and elective admissions, were more common in cases than in controls. Emergency admissions were more common than elective admissions in cases, with the opposite being true in controls. All the studied types of hospital in-patient admission were more common in cases than in controls. The highest OR was for self-harm emergency admissions (OR = 94.9; 95% CI 63.5–141.9). More females (518/1131 (45.9%); OR = 4.0 (95% CI 3.6–4.4)) than males (1244/3999 (31.2%); OR = 3.3 (95% CI 3.1–3.5)) had hospital admissions in the year before their index date. The same was true for all studied hospital admissions except for very rare contacts (<2%), drugs misuse emergency admissions, and alcohol and drugs misuse elective admissions. ORs for females were higher than for males, particularly for alcohol misuse emergency admissions (OR = 45.4 (95% CI 22.4–90.8) *v*. OR = 16.6 (95% CI 12.7–21.7)). Confidence intervals were wide because of the small number of controls for these contacts (<0.5%). Injury and poisoning emergency admissions were relatively common in both genders (females: 221/1131 (19.6%); males: 488/1244 (12.3%)) and showed higher ORs in females (OR = 19.8 (95% CI 15.1–26.0) *v*. OR = 10.4 (95% CI 9.1–12.0)).

**Table 2:** Type of hospital admissions in the year before index date

	Cases (*N* = 5130)		Controls (*N* = 25 650)		OR	95% CI
	*n* (%)	95% CI	*n* (%)	95% CI		
Any contact	1762 (34.4)	33.1–35.7	3406 (13.3)	12.9–13.7	3.5	3.3–3.6
Mental health specialty	498 (9.8)	9.0–10.6	49 (0.2)	0.2–0.3	53.3	39.8–71.3
Emergency admissions by diagnosis						
Any	1461 (28.5)	27.3–29.8	1491 (5.9)	5.6–6.2	6.4	6.0–6.9
Mental health	898 (17.6)	16.5–18.6	282 (1.1)	1.0–1.3	18.6	16.3–21.2
Common mental disorder	497 (9.7)	9.0–10.6	90 (0.4)	0.3–0.5	30.1	24.1–37.6
Injury and poisoning	709 (13.9)	13.0–14.8	315 (1.3)	1.2–1.4	12.3	10.9–13.9
Accidents	243 (4.8)	4.2–5.4	230 (0.9)	0.8–1.1	5.2	4.5–6.0
Accidental hanging and poisoning	82 (1.6)	1.3–2.0	<13 (0.1)	0.1–0.1	42.5	21.9–82.3
Self-harm	471 (9.2)	8.5–10.1	26 (0.2)	0.1–0.2	94.9	63.5–141.9
Alcohol misuse	278 (5.5)	4.9–6.1	75 (0.3)	0.3–0.4	20.1	15.7–25.7
Drugs misuse	97 (1.9)	1.6–2.4	<29 (0.2)	0.1–0.2	17.3	11.6–25.6
Elective admissions by diagnosis						
Any	608 (11.9)	11.0–12.8	2187 (8.6)	8.2–8.9	1.5	1.4–1.6
Mental health	134 (2.7)	2.3–3.1	110 (0.5)	0.4–0.6	6.1	5.0–7.5
Common mental disorder	56 (1.1)	0.9–1.5	31 (0.2)	0.1–0.2	9.2	6.3–13.3
Alcohol misuse	24 (0.5)	0.4–0.7	<14 (0.1)	0.1–0.1	11.3	5.9–21.7
Drugs misuse	<12 (0.3)	0.2–0.5	<5 (0.1)	0.1–0.1	13.3	4.1–42.8

a. Results from those with Patients Episode Database for Wales data (i.e. the full study population). Odds ratios (OR) adjusted for deprivation. *P* < 0.001 for all results.

In the year before the index date, 1409/3861 cases (36.5%) and 5146/19 305 controls (26.7%) had one or more out-patient contacts (OR = 1.6; 95% CI 1.5–1.7). In this period, 452/3861 cases (11.8%) and 224/19 305 controls (1.2%) had a mental health specialty out-patient contact (OR = 11.2; 95% CI 9.7–13.0). Stratifying by gender, 405/835 of females (48.6%) and 1004/3026 of males (33.2%) had an out-patient contact, with OR = 1.8 (95% CI (1.7–2.0) and OR = 1.6 (95% CI 1.5–1.6) respectively. Similarly, 154/835 of females (18.5%) and 298/3026 of males (9.9%) had a mental health specialty out-patient contact, with OR = 19.3 (95% CI 14.0–26.7) and OR = 9.2 (95% CI 7.8–10.9) respectively.

### Contacts with primary and secondary healthcare: analysis of linked data across all healthcare settings

#### Use of healthcare services in the year leading to the index date

Table 3 shows full results for cases and controls with healthcare contacts across services, with gender stratification in supplementary Table 7. Overall, more females and cases had contacts with healthcare services in the time leading to their index date, particularly in periods closer to this date. The general practice was the most common point of contact (one-quarter of cases in the week before the index date and two-thirds in the month before the index date). ORs were highest for emergency department contacts: OR = 10.1 (95% CI 7.2–14.0) and OR = 5.5 (95% CI 4.8–6.3) in the week and month before the index date respectively. Indeed, 4.5 and 16.6% of all those who died by suicide had been seen in the emergency department in the week and month before the index date respectively. This was followed by hospital admissions, general practice and hospital out-patient contacts. Females contacted healthcare services more than males: 172/359 (48.0%) *v*. 367/1362 (27.0%) in the week before the index date, and 327/359 (91.1%) *v*. 931/1362 (68.4%) in the month before. ORs for females were higher than for males, particularly for emergency department contacts (OR = 20.0 (95% CI 9.3–42.7) *v*. OR = 7.9 (95% CI 5.4–11.4) in the week before the index date; and OR = 8.4 (95% CI 6.2–11.2) *v*. OR = 4.8 (95% CI 4.1–5.6) in the month before).

**Table 3:** Type of healthcare setting contacted before the index date for the fully linked study population across healthcare settings^a^

	Cases (*N* = 1721)		Controls (*N* = 8605)		OR	95% CI
	*n* (%)	95% CI	*n* (%)	95% CI		
Any healthcare setting						
1 week	539 (31.4)	29.2–33.6	1339 (15.6)	14.9–16.4	2.6	2.4–2.8
1 month	1258 (73.1)	71.0–75.2	4540 (52.8)	51.8–53.9	2.7	2.6–3.0
1 year	1536 (89.3)	87.7–90.7	6525 (75.9)	75.0–76.8	2.8	2.5–3.1
General practice						
1 week	444 (25.8)	23.8–28.0	1182 (13.8)	13.1–14.5	2.3	2.1–2.5
1 month	1175 (68.3)	66.1–70.5	4332 (50.4)	49.3–51.4	2.4	2.2–2.5
1 year	1460 (84.9)	83.1–86.5	6209 (72.2)	71.2–73.1	2.3	2.1–2.5
Emergency department						
1 week	77 (4.5)	3.6–5.6	42 (0.5)	0.4–0.7	10.1	7.2–14.0
1 month	285 (16.6)	14.9–18.4	312 (3.7)	3.3–4.1	5.5	4.8–6.3
1 year	712 (41.4)	39.1–43.8	1426 (16.6)	15.9–17.4	3.5	3.3–3.8
Hospital in-patients						
1 week	68 (4)	3.2–5.0	66 (0.8)	0.7–1.0	5.8	4.4–7.7
1 month	223 (13)	11.5–14.7	303 (3.6)	3.2–4.0	4.2	3.6–4.8
1 year	569 (33.1)	30.9–35.4	1183 (13.8)	13.1–14.5	3.1	2.9–3.4
Hospital out-patients						
1 week	49 (2.9)	2.2–3.8	139 (1.7)	1.4–2.0	1.8	1.5–2.2
1 month	288 (16.8)	15.1–18.6	870 (10.2)	9.5–10.8	1.8	1.6–2.0
1 year	714 (41.5)	39.2–43.9	2509 (29.2)	28.3–30.2	1.8	1.7–1.9

a. Results from those with data across all settings in the year before index date (i.e. those with an index date in 2010 or later and General Practice Database data available in the year before index date). Odds ratios (OR) adjusted for deprivation. *P* < 0.001 for all results.

The last point of contact was: general practice for 1226/1721 (71.3%; 95% CI 69.1–73.4%); emergency department for 133/1721 (7.8%; 95% CI 6.6–9.1%); hospital admission for 100/1721 (5.9%; 95% CI 4.9–7.1%); and hospital out-patients for 115/1721 (6.7%; 95% CI 5.6–8.0%). This was similar for both genders. Of the 213 admitted to hospital and/or seen in out-patients, 60/213 (28.2%; 95% CI 22.6–34.5%) were in a mental health specialty. A mental health diagnosis was recorded at the last contact before index date for 167/1721 cases (9.8%; 95% CI 8.4–11.2%), injury and poisoning for 77/1721 cases (4.5%; 95% CI 3.6–5.6%), accidents for 43/1721 cases (2.5%; 95% CI 1.9–3.4%) and self-harm for 42/1721 cases (2.5%; 95% CI 1.9–3.3%). The same pattern was observed in both genders, with slightly higher rates in females (mirroring the higher rates of females in contact with health services), with no significant difference between the two across all the studied types of contact.

#### Trajectories of health service use in the year prior to the index date

**Fig. 1:**
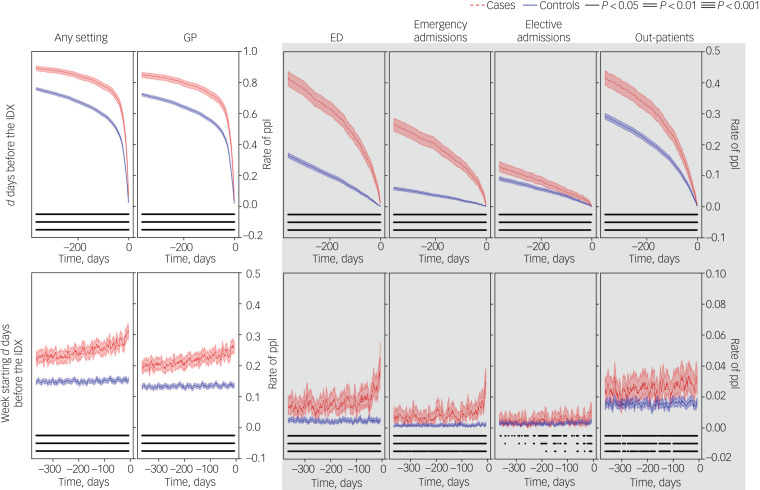
Rate of people (ppl) who died by suicide (cases) and controls with any type of contact at each healthcare setting in the 12 months before the index date (the date of death or the start date of a healthcare contact during which the death occurred). Contacts in the 1–365 days before the index date (IDX) (top) and in the week starting 7–365 days before the index date (bottom). Columns correspond to contacts with any setting (Any), primary care/general practice (GP), emergency department (ED), emergency hospital admissions (H-E), planned hospital admissions (H-P) and out-patients (OP). Graphs outside and inside the shaded area share *y*-axis limits. *P*-values correspond to conditional logistic regression adjusted for deprivation.

A continuous, follow-back representation of the frequency of cases and controls contacting healthcare services in the *d* days before the index date can be seen in the upper row of Fig. 1. The frequency of individuals contacting healthcare services mostly follows a logarithmic decaying trend more accentuated in cases than in controls, with ORs between 2.5 and 3.0 and always statistically significant. The frequency of individuals with a general practice contact follows a similar pattern. In emergency department contacts, this decreases linearly and more rapidly before sharply dropping in the weeks before the index date for cases, while it remains mostly linear during the whole period for controls, with a slope close to that for cases. A similar pattern can be seen for emergency hospital admissions, albeit with greater slope difference between cases and controls.

An additional continuous, follow-back representation of cases and controls contacting healthcare services in the week starting at day *d* before the index date can be seen in the lower row of Fig. 1. At any given week in the year before the index date, the frequency of cases contacting healthcare services (any) was higher than that for controls. We observed mostly linear trends, increasing for cases – particularly in general practice contacts – and flat for controls. Two exceptions were emergency department contacts and emergency hospital admissions, which exhibited a sharp increase in the weeks before the index date.

The continuous follow-back representation for mental health and self-harm contacts can be seen in supplementary Fig. 2. The frequency of cases with mental health contacts in the *d* days before the index date decayed linearly first and abruptly close to the index date compared with controls, and the frequency of cases with mental health contacts in the week starting *d* days before the index date had the equivalent upward behaviour. At any given week in the year before the index date, the frequency of cases with a mental health contact was higher than that for controls. The frequency of cases with self-harm contacts in the *d* days before the index date decayed mostly linearly, and the frequency of cases with self-harm contacts in the week starting *d* days before the index date was flat until peaking in the weeks before the index date. In this case, the difference between the frequencies for cases and controls with a self-harm contact at any given week in the year before the index date was not always statistically significant.

### Sensitivity analysis

Of the study population, 4091/5130 (79.7%) had their deaths coded as intentional self-harm/suicide (ICD-10 codes X60–84) and 1039/5130 (20.2%) were ‘deaths of undetermined intent’ (Y10–34, Y87 and Y87.2; excluding Y33.9 before 2007). A sensitivity analysis including only those who died by suicide showed no significant differences to any of our findings using the full study population. Supplementary Table 8 shows the demographics for the full sensitivity study population and for those with data coverage across all settings (equivalent to supplementary Table 4). Supplementary Table 9 shows the type of healthcare setting contacted before the index date for the fully linked sensitivity study population across healthcare settings (equivalent to Table 3).

## Discussion

We found high frequencies of contact with healthcare services in those who died by suicide, particularly in close proximity to their death. Almost four out of five of those who died by suicide had contact with healthcare services in the month before their death. As close as 1 week before, almost one in three had a contact with healthcare services, a rate twice that for the general population. The majority of these contacts were in general practice settings: 26% of cases had a general practice contact in the week before they died. A general practice setting was also the most common last point of contact (for 71% of cases). Males consistently had fewer contacts than females across all settings, but still three-quarters of males who died by suicide had at least one healthcare contact in the month before they died. Conversely, OR estimates for females with healthcare contacts were generally higher than for males in comparison with the general population across all settings and time points. Thus, females show a larger change in healthcare contact patterns during a crisis than males.

Our longitudinal analysis by week showed that those who died by suicide were more likely to contact healthcare services in any given week in the year before their death than the general population. The likelihood of contact increased with time closer to their death. This was particularly marked for emergency department contacts and emergency hospital admissions, whose likelihood of contact increased dramatically in the weeks before the index date. This may reflect the well-recognised increase in risk of suicide seen in the period shortly after hospital attendance for self-harm.^17^ At earlier time points, many contacts represent repeated visits (i.e. not the last visit before the index date), except for elective hospital admissions, as evidenced by the comparison between the cumulative and weekly graphs. There does appear to be an escalating build-up of help-seeking contacts over time leading to an acute crisis. Cues flagging those at risk of suicide seem to accumulate in the year before they die by suicide, predominantly in the weeks before such crisis.

We found that the association between risk of suicide and mental health diagnoses, alcohol misuse, drugs misuse and self-harm was stronger in secondary care settings, particularly the emergency department and hospital. This is possibly related to the severity of such contacts. For example, even though self-harm emergency hospital admissions were rare events, they were almost 100 times more likely in cases than in controls. However, ‘injury and poisoning’ and ‘accidental hanging and poisoning’ in hospital admissions may potentially be miscoded, resulting in an underestimation of self-harm contacts. Over 1 in 4 of those who took their own lives attended emergency departments because of injury or poisoning in the previous year.

### Comparison with other studies

Our estimates related to contacts with services were similar to those found in the literature since 1975.^4–8,18^ Differences in the type, coverage and quality of the data used and in the way the same variables are defined, as well as social and demographic differences, may affect results and explain any variability. Previous studies have highlighted general practice as a point of contact in the year before death by suicide for many patients (range 49–92%) but this focus on general practice neglects contacts with other sectors of the healthcare system and is lacking in the comprehensive assessment of healthcare contacts in our study.^4,5^ These high levels of contact in part reflect the high levels of contact that these patients have with general practices, and our study puts these contacts into context by comparison with general population controls. An increase in the likelihood of contact closer to death in those who die by suicide was suggested previously, but no comparison was made with contact patterns in the general population.^19^ Our finding that 41% of those who died by suicide were in contact with an emergency department in the year before their index date is consistent with Gairin et al's,^20^ potentially indicating little improvement in the management of those presenting in this setting since 1997. The association between risk of suicide and mental health diagnoses, alcohol misuse, drugs misuse and self-harm has been extensively reported elsewhere, generally on the basis of analysis of single healthcare settings.^21,22^

### Strengths and limitations

Linking data at a person level across healthcare settings and performing a time-continuous analysis, we have been able to present a detailed picture of the type and pattern of health service contacts in the year before their death for those who died by suicide, compared with general population controls, in routinely collected administrative data covering a whole population over 17 years. To reduce bias, controls were matched on gender and week of birth and alive at the time of death of the case. By linking to demographic data, we adjusted our results for deprivation. The demography of Wales is characterised by relatively high levels of deprivation and population sparsity compared with other parts of the UK.^23^ These factors are known to affect access to services and mental health outcomes. Having said that, our findings on contacts with healthcare settings were similar to studies conducted in other regions.^5^ The size of our study allowed us to look at male and female contacts separately.

We identified and removed from the analysis any healthcare contact where the individual died during that contact or during a resulting admission. Most published studies use death date as the index date, which may bias results.^4,5^ For example, individuals who die during a 1-month hospital stay cannot contact a general practice, emergency department or out-patients in that period, thus artificially lowering the rate for these settings in the month before the index date. Similarly, if a diagnosis is recorded for the first time during the contact leading to death, it may not be correct to consider that diagnosis as a risk factor, since it would have presented after the last opportunity for intervention (outside in-patient suicide prevention factors). Thus, our definition of index date represents the last date at which patients had an opportunity to contact any healthcare service. However, we do note that during an admission, healthcare services have an opportunity to identify those at risk of suicide on the basis of history of previous contacts with such services.

Limitations of the use of routinely collected data for research purposes have been reported elsewhere.^12,24^ We reduced misclassification bias by using validated code lists, developed and updated with the help of expert clinicians to identify our study cohort and outcomes of interest. The use of a case–control study design where both groups experienced the same ascertainment issue somewhat alleviated missing variables and missing data biases.

Since secondary care and other contacts are communicated to general practices, we were unable to guarantee that only face-to-face or telephone general practice contacts were included in the analysis. This is similar to other studies of this type^25^ and may have inflated results of contacts in general practice settings. We alleviated this by excluding administrative codes such as ‘letter from emergency department’ or ‘patient seen in emergency department’ and associated diagnoses, keeping telephone and face-to-face contacts with any member of the general practice team.

Health data coverage was not always available for the whole study population or study period. We circumvented this limitation by running independent analyses with each of the data-sets (health settings). All subpopulations based on data coverage limitations in the linked data-set had comparable demographic distributions to the full population, implying that results were generalisable.

### Implications for research, policy and practice

This study provides an opportunity to identify better strategies for suicide prevention by highlighting the importance of patients’ patterns of contacts with services. We may be missing opportunities to help those at risk that exist for over a year before they die. We have highlighted the need for general practices, who are often the last point of contact, to consider their patients’ contacts with other health services as part of their assessment. In the UK, contacts with secondary care services are notified to general practices. While general practice has the potential to support those at risk of suicide through therapeutic conversations and suicide risk assessment there are challenges that should be acknowledged in this setting. These include time constraints, workload, training and a lack of integration with specialist mental health services and broader public health suicide prevention initiatives. Addressing these barriers, given the importance of this setting as a point of contact among those who go on to take their own lives, is vital to promote effective enhanced system-level approaches.^25^

It does appear that help-seeking occurs in those at risk of suicide and escalates, particularly in the weeks before death and in females. Although patients do not always disclose suicidal ideation or mental health problems, there are markers that should be acted on, such as a previous suicide attempt, sleep disturbance and chronic pain. This study highlights the importance of evaluating patients following emergency department or hospital discharge, particularly when for mental health-related problems, self-harm, alcohol or substance misuse, and of ensuring follow-up. General practice contacts and emergency hospital admissions due to alcohol and drugs misuse were particularly associated with risk of suicide in females, and intervention with females presenting to services with these problems at scale may have a proportionate impact on suicide prevention efforts.

Despite these existing cues, identifying those at risk of suicide is complex. Predictive models and tools such as those used in other specialties^26^ or using traditional epidemiological techniques are not powerful enough in this context.^27^ There may be promise in exploring advance machine-learning techniques to help progress our understanding of suicide, identifying novel risk factors and/or new interaction models.^28^ These techniques could also result in clinical decision support tools, which may help suicide prevention efforts, especially in general practice settings. Given the low incidence of suicide, even in clinical populations, predicting outcomes is likely to be an unrealistic goal. However, these machine-learning-based strategies may be a useful adjunct to clinical management.^29^

Health professionals without mental healthcare training may not routinely ask about suicidal thoughts even with patients who have a history of attempted suicide,^30^ and raising awareness of suicide risk and improving training of such staff may help them to ask these questions, put safety plans in place and signpost patients to other services. These opportunities for suicide prevention highlight the importance of adherence to guidance following presentation to services with self-harm,^31^ particularly comprehensive psychosocial assessments, supported by initiatives such as the National Collaborating Centre for Mental Health's core competency frameworks for self-harm,^32^ creating a core curriculum for health service staff.

## Data Availability

The raw data used in this study are available on application to the SAIL Databank via their usual governance processes (www.saildatabank.com). Derived data supporting the findings of this study are available from the corresponding author (A.J.) on request.

## Supplementary material

For supplementary material accompanying this paper visit https://doi.org/10.1192/bjp.2020.137. click here to view supplementary material
