# Supplementary material for: Contacts with primary and secondary healthcare prior to suicide: case–control whole-population-based study using person-level linked routine data in Wales, UK, 2000–2017
Source: Br J Psychiatry. 2020 Aug 3;217(6):717–24. doi: 10.1192/bjp.2020.137 (PMC7705668; doi:10.1192/bjp.2020.137)
Supplement: Supplementary file 1 [file S0007125020001373sup001.zip › S0007125020001373sup001.docx]

**Contacts with primary and secondary health care prior to suicide: a case-control population-based study using person-level linked routine data.**

Ann John, Marcos DelPozo-Banos, David Gunnell, Michael Dennis Jonathan Scourfield, David V Ford, Nav Kapur, Keith Lloyd

**SUPPLEMENTARY MATERIAL**

**Supplementary Table 1:** Datasets used in this analysis.

| **Database** | **Description** | **Coverage** |
| --- | --- | --- |
| Welsh Demographic Service | An administrative register of all individuals in Wales that use NHS services, containing anonymised demographics and GP practice registration history with anonymised residential data | The whole population of Wales during the study period |
| Office for National Statistics - Deaths | Death register of all deaths and causes in Wales, coded using International Classification of Diseases (ICD), version 10 codes | The whole population of Wales during the study period |
| General Practice Database (GPD) | Primary care records with diagnoses, symptoms, investigations, prescribed medication, referrals, coded hospital contacts, and test results coded using Read Codes v2 | 77% (333/432) of all general practices in Wales |
| Emergency Department Data Set | Administrative and clinical information (general reason for attendance and attendance group to identify types of contacts) for all NHS Wales Accident and Emergency department attendances | The whole population of Wales from 2009 |
| Patient Episode Database for Wales | Clinical information (specialty and diagnoses) of all NHS Wales hospital admissions (inpatient and day cases) – diagnostic information coded using ICD-10 codes | The whole population of Wales during the study period |
| Outpatient Dataset | Attendance information for all NHS Wales hospital outpatient appointments. Since diagnostic information was not available, we used consultant main specialty and treatment specialty to identify mental health contacts | The whole population of Wales from 2004 |

**Supplementary Table 2:** Variables used in this analysis.

| **Extracted variables** | |
| --- | --- |
| **Name** | **Queried datasets** |
| Contacts | |
| Any contact (a recorded entry) | GPD (excluding administrative codes and associated diagnoses such as ‘letter from ED’, but including telephone and face-to-face contacts with any member of the primary care team); Emergency Department Dataset; Patient Episode Database for Wales; Outpatients Dataset |
| Mental health specialty (psychiatric or child and adolescent psychiatric speciality) | GPD (Read code denoting contacts with mental health specialty services); Patient Episode Database for Wales; Outpatients Dataset (consultant main specialty and treatment specialty visited) |
| Diagnoses | |
| Non-mental health | GPD (Read codes with a capital letter root other than ‘E’ comprising mainly ‘physical’ diagnoses) |
| Mental health | GPD (Read codes with root ‘E’ plus relevant symptom codes for common mental disorders); Emergency Department Dataset; Patient Episode Database for Wales |
| Common mental disorder (depression and anxiety) | GPD; PEDW |
| Injury & poisoning (excluding self-harm) | GPD; Emergency Department Dataset; Patient Episode Database for Wales |
| Accidents | GPD; Emergency Department Dataset; Patient Episode Database for Wales |
| Accidental hanging & poisoning | GPD; Patient Episode Database for Wales |
| Self-harm | GPD; Emergency Department Dataset; Patient Episode Database for Wales |
| Alcohol misuse | GPD; Emergency Department Dataset; Patient Episode Database for Wales |
| Drugs misuse | GPD; Emergency Department Dataset; Patient Episode Database for Wales |
| Prescriptions | |
| Any prescription | GPD (Read code with a lower case letter root) |
| Opiates | GPD |
| Psychotropics (antidepressants, antipsychotics, anxiolitics, and hypnotics) | GPD |

**Supplementary Table 3:** List of Read Codes for GPD and ICD-10 codes for PEDW used to identify types of contacts. Validated by previous research (references in table) and extended or created with the help of expert clinicians.

| **Read Codes** | **ICD-10** |
| --- | --- |
| **Accidents** | |
| SS, T0-T9, TA-TH, TJ, U0, U1 | V, W, X1-X5 |
| **Accidental hanging and poisoning** | |
| TH..., TH0, T8, T9, TF3, U14.., U1A, U140-U142 Except: T90.., T900, TH00-TH05, TH0y, TH0z, U1A9 | W75, W76, X4 Except: X45 |
| **Alcohol misuse^A, B^** | |
| 7P221, C1505, F11x0, F1440, F3941, G8523, J6708, J6710, U60H3, ZV6D6, 1365, 1366, 136K, 136P, 136Q, 136R, 136S, 136T, 136W, 13Y8, 1462, 1B1c, 388j, 38Dz, 8BA8, 8G32, 8H35, 8H7p, 8HkG, 8HkJ, 8IAF, 8IAJ, 8IAt, 8IEA, 9N2o, 9NN2, C253, C25X, E250, Eu10, F25B, F375, G555, J153, J610, J611, J612, J613, J617, PK80, SLH3, TJH3,, 66e, 9k1, E01, E23, du1, du5 Except: E0112, 9k15, 9k16, 9k17, 9k18, 9k19 | E244, G312, G621, G721, I426, K292, K852, K860, Y573, Z502, Z714, Z721, F10, K70 |
| **Common mental disorder^C, D^** | |
| Eu341, Eu930, Eu931, Eu932, 1B12, 1B13, 1B17, 1B1U, 1B1V, 2257, 2258, 2259, 225J, E112, E113, E118, E135, E200, E202, E204, E291, E2D0, Eu32, Eu33, Eu40, Eu41. R2y2, 1BP, 1BQ, 1BT, 1BU, E2B Except: E1124, E1134, Eu323, Eu328, Eu329, Eu32A, Eu333 | F32, F33, F341, F40, F41 Except: F323, F333 |
| **Drugs misuse^E, F^** | |
| 7P220, 9N1yJ, R10B0, R10B1, R10B2, R10B4, ZV6D7, 13c, 1TE, 1TF, 8AA, 8FB, 8Hq, 9G2, 9HC, 9k5, E02, E24, E25, Eu1, djc, du..., du2, du4, 146C, 1P30, 677T, 8B23, 8BAW, 8BAX, 8BAc, 8CR9, 8H7x, 8HHL, 8HHd, 8HHe, 8Hh1, 8HkF, 8Hl5, 8Hl6, 8I2N, 9N6a, 9N6b, 9NdN, 9No5, L183, SL97, T800, dj36, dj37, dj38, dj3D, dj3E, dj3F, dj3G, dj3u, dj3v, 1V Except: SL971, 13cP, 1V23, 1V25, 1V26, 1V50, 1V60, 1V61, 1V62, 1V63, 8FB0, 9HC0, 9HC1, 9HC5, 9HC8, E023, E250, E251, Eu10, Eu17, du24 | F1, R782, R783, Z503, Z715, Z722 Except: F10, F17 |
| **Injury and poisoning^E, F^** | |
| S, T, U Except: SL, U72, SL90, SLHz, U720, SS | S, T |
| **Mental health** | |
| E | F |
| **Physical health** | |
| A, B, C, D, F, G, H, J, K, L , M, N, R, Z |  |
| **Self-harm^G^** | |
| SL, U…, U0…, U72.., 14K1, SL90, SLHz, U720, TK, TN, TP, Tz, U2, ZX, U00, U01, U02, U41, U44, U45, U4BExcept: U209, U409, U02z4, U02z5, TK, TK, U02z6, U02z8, U02zz | X60-X84, Y10-Y34, Y870, Y872 Except: X65, Y15 |
| **Prescriptions** | |
| a-z Except: t, v, w, x, z |  |
| **Prescription of opiates** | |
| 8B23, 8B2M, 8B2N, 8B2P, 8B2Q, 8B2R, 8BE, a81, cg1, cg3, cg5, cg7, dia, dib, dic, did6, dj, j28O, j28V, j2pT, o4, oa2N, oa2J Except: dia8A-diaF, diaH, diaM, diaN, diaQ, diaR, diaV-diaZ, diaa, diac, diaf, diag, diaj, dial, diau. diay, diaz, dib1, dib2, dib4-dib7, dibC, dibL, dibN, dibO, dibP, dibT, dibU, dibV, dibY, dibZ, dibi, dibk, dibl, dibm, dibq, dibr, diby, dic1, dic2, dic6, dicB-dicI, dicP, dicQ, dicT-dicX, dicZ, dicw, djm, o43, o4a, cg7c, dj3K, dj3L, dj3M, djkb, djkc, djkd, djyJ, djyK, djyL, o4e4-o4e8 |  |
| **Prescription of psychotropics^H^** | |
| d1, d2, d4, d5-d9, da, gde Except: d19, d1e, d4y, da8, d4v9, drvR, d4w9, d4wR, d915 |  |

1. McKenzie K, Harrison JE, McClure RJ. Identification of alcohol involvement in injury-related hospitalisations using routine data compared to medical record review. Australian and New Zealand journal of public health. 2010;34(2):146-52.
2. Carr MJ, Ashcroft DM, Kontopantelis E, While D, Awenat Y, Cooper J, et al. Premature death among primary care patients with a history of self-harm. The Annals of Family Medicine. 2017;15(3):246-54.
3. John A, McGregor J, Fone D, Dunstan F, Cornish R, Lyons RA, et al. Case-finding for common mental disorders of anxiety and depression in primary care: an external validation of routinely collected data. BMC medical informatics and decision making. 2016;16(1):35.
4. WHO. The ICD-10 classification of mental and behavioural disorders : clinical descriptions and diagnostic guidelines.: World Health Organization; 1992 [Available from: <https://apps.who.int/iris/handle/10665/37958>.
5. Quan H, Sundararajan V, Halfon P, Fong A, Burnand B, Luthi J-C, et al. Coding algorithms for defining comorbidities in ICD-9-CM and ICD-10 administrative data. Medical care. 2005:1130-9.
6. Thompson A, Shaw M, Harrison G, Ho D, Gunnell D, Verne J. Patterns of hospital admission for adult psychiatric illness in England: analysis of Hospital Episode Statistics data. Br J Psychiatry. 2004;185:334-41.
7. Marchant A, Turner S, Balbuena L, Peters E, Williams D, Lloyd K, et al. Self-harm presentation across healthcare settings by sex in young people: an e-cohort study using routinely collected linked healthcare data in Wales, UK. Archives of disease in childhood. 2019:archdischild-2019-317248.
8. John A, Marchant A, Fone D, McGregor J, Dennis M, Tan J, et al. Recent trends in primary-care antidepressant prescribing to children and young people: an e-cohort study. Psychological medicine. 2016;46(16):3315-27.

**Supplementary Table 4** **(part 1/3):** Demographic information of the study population. Full study population and those with GPD data available in the year before the IDX. Numbers are counts (N), percentage and 95% CI.

|  | **Study population** | | | |  | **Study population with GPD data available in the year before the IDX** | | | |
| --- | --- | --- | --- | --- | --- | --- | --- | --- | --- |
|  | N | **Cases** | N | **Controls** |  | N | **Cases** | N | **Controls** |
|  | % | [95% CI] | % | [95% CI] |  | % | [95% CI] | % | [95% CI] |
| **Total** | **5130** |  | **25650** |  |  | **3504** |  | **17520** |  |
| **Males/Females** | | | | | | | | | |
| Females | 1131 |  | 5655 |  |  | 779 |  | 3895 |  |
|  | 22.1 | [21.0, 23.3] | 22.1 | [21.6, 22.6] |  | 22.3 | [20.9, 23.7] | 22.3 | [21.7, 22.9] |
| Males | 3999 |  | 19995 |  |  | 2725 |  | 13625 |  |
|  | 78 | [76.8, 79.1] | 78 | [77.5, 78.5] |  | 77.8 | [76.4, 79.2] | 77.8 | [77.2, 78.4] |
| **Age group** | | | | | | | | | |
| Children & young people: [10, 24] y.o. | 571 |  | 2856 |  |  | 373 |  | 1866 |  |
|  | 11.2 | [10.3, 12.1] | 11.2 | [10.8, 11.6] |  | 10.7 | [9.7, 11.8] | 10.7 | [10.3, 11.2] |
| Adults: [25, 64] y.o. | 3742 |  | 18717 |  |  | 2538 |  | 12697 |  |
|  | 73 | [71.8, 74.2] | 73 | [72.5, 73.6] |  | 72.5 | [71.0, 73.9] | 72.5 | [71.9, 73.2] |
| Older population: 64+ y.o. | 817 |  | 4077 |  |  | 593 |  | 2957 |  |
|  | 16 | [15.0, 17.0] | 15.9 | [15.5, 16.4] |  | 17 | [15.8, 18.3] | 16.9 | [16.4, 17.5] |
| **WIMD deprivation** | | | | | | | | | |
| Least deprived - 1 | 681 |  | 5436 |  |  | 508 |  | 3783 |  |
|  | 13.3 | [12.4, 14.3] | 21.2 | [20.7, 21.7] |  | 14.5 | [13.4, 15.8] | 21.6 | [21.0, 22.3] |
| 2 | 841 |  | 4841 |  |  | 540 |  | 3215 |  |
|  | 16.4 | [15.5, 17.5] | 18.9 | [18.4, 19.4] |  | 15.5 | [14.3, 16.7] | 18.4 | [17.8, 19.0] |
| 3 | 956 |  | 5253 |  |  | 683 |  | 3587 |  |
|  | 18.7 | [17.6, 19.8] | 20.5 | [20.0, 21.0] |  | 19.5 | [18.3, 20.9] | 20.5 | [19.9, 21.1] |
| 4 | 1075 |  | 5035 |  |  | 721 |  | 3430 |  |
|  | 21 | [19.9, 22.1] | 19.7 | [19.2, 20.2] |  | 20.6 | [19.3, 22.0] | 19.6 | [19.0, 20.2] |
| Most deprived - 5 | 1264 |  | 5051 |  |  | 911 |  | 3490 |  |
|  | 24.7 | [23.5, 25.9] | 19.7 | [19.3, 20.2] |  | 26 | [24.6, 27.5] | 20 | [19.4, 20.6] |
| **Area morphology** | | | | | | | | | |
| Urban | 3241 |  | 17532 |  |  | 2363 |  | 12117 |  |
|  | 63.2 | [61.9, 64.5] | 68.4 | [67.8, 69.0] |  | 67.5 | [65.9, 69.0] | 69.2 | [68.5, 69.9] |
| Rural | 1576 |  | 8084 |  |  | 1000 |  | 5388 |  |
|  | 30.8 | [29.5, 32.0] | 31.6 | [31.0, 32.1] |  | 28.6 | [27.1, 30.1] | 30.8 | [30.1, 31.5] |

**Supplementary Table 4 (part 2/3):** Demographic information of the study population. Those with ED data and Outpatient data available in the 1-year before the IDX. Numbers are counts (N), percentage and 95% CI.

|  | **Study population with ED data available in the year before the IDX** | | | |  | **Study population with Outpatient data available in the year before the IDX** | | | |
| --- | --- | --- | --- | --- | --- | --- | --- | --- | --- |
|  | N | **Cases** | N | **Controls** |  | N | **Cases** | N | **Controls** |
|  | % | [95% CI] | % | [95% CI] |  | % | [95% CI] | % | [95% CI] |
| **Total** | **2476** |  | **12380** |  |  | **3861** |  | **19305** |  |
| **Males/Females** | | | | | | | | | |
| Females | 521 |  | 2605 |  |  | 835 |  | 4175 |  |
|  | 21.1 | [19.5, 22.7] | 21.1 | [20.4, 21.8] |  | 21.7 | [20.4, 23.0] | 21.7 | [21.1, 22.3] |
| Males | 1955 |  | 9775 |  |  | 3026 |  | 15130 |  |
|  | 79 | [77.4, 80.6] | 79 | [78.3, 79.7] |  | 78.4 | [77.1, 79.7] | 78.4 | [77.8, 79.0] |
| **Age group** | | | | | | | | | |
| Children & young people: [10, 24] y.o. | 255 |  | 1275 |  |  | 401 |  | 2006 |  |
|  | 10.3 | [9.2, 11.6] | 10.3 | [9.8, 10.9] |  | 10.4 | [9.5, 11.4] | 10.4 | [10.0, 10.9] |
| Adults: [25, 64] y.o. | 1814 |  | 9077 |  |  | 2826 |  | 14138 |  |
|  | 73.3 | [71.5, 75.0] | 73.4 | [72.6, 74.1] |  | 73.2 | [71.8, 74.6] | 73.3 | [72.7, 73.9] |
| Older population: 64+ y.o. | 407 |  | 2028 |  |  | 634 |  | 3161 |  |
|  | 16.5 | [15.1, 18.0] | 16.4 | [15.8, 17.1] |  | 16.5 | [15.3, 17.7] | 16.4 | [15.9, 17.0] |
| **WIMD deprivation** | | | | | | | | | |
| Least deprived - 1 | 336 |  | 2575 |  |  | 528 |  | 4040 |  |
|  | 13.6 | [12.3, 15.0] | 20.8 | [20.1, 21.6] |  | 13.7 | [12.7, 14.8] | 21 | [20.4, 21.6] |
| 2 | 420 |  | 2275 |  |  | 632 |  | 3636 |  |
|  | 17 | [15.6, 18.5] | 18.4 | [17.8, 19.1] |  | 16.4 | [15.3, 17.6] | 18.9 | [18.3, 19.4] |
| 3 | 476 |  | 2566 |  |  | 749 |  | 3978 |  |
|  | 19.3 | [17.8, 20.9] | 20.8 | [20.1, 21.5] |  | 19.4 | [18.2, 20.7] | 20.7 | [20.1, 21.2] |
| 4 | 515 |  | 2442 |  |  | 799 |  | 3788 |  |
|  | 20.8 | [19.3, 22.5] | 19.8 | [19.1, 20.5] |  | 20.7 | [19.5, 22.1] | 19.7 | [19.1, 20.2] |
| Most deprived - 5 | 566 |  | 2504 |  |  | 928 |  | 3838 |  |
|  | 22.9 | [21.3, 24.6] | 20.3 | [19.6, 21.0] |  | 24.1 | [22.8, 25.5] | 19.9 | [19.4, 20.5] |
| **Area morphology** | | | | | | | | | |
| Urban | 1536 |  | 8483 |  |  | 2455 |  | 13215 |  |
|  | 62.1 | [60.2, 64.0] | 68.6 | [67.7, 69.4] |  | 63.6 | [62.1, 65.1] | 68.5 | [67.8, 69.2] |
| Rural | 777 |  | 3879 |  |  | 1181 |  | 6065 |  |
|  | 31.4 | [29.6, 33.3] | 31.4 | [30.6, 32.2] |  | 30.6 | [29.2, 32.1] | 31.5 | [30.8, 32.1] |

**Supplementary Table 4 (part 3/3):** Demographic information of the study population. Those with fully linked data (i.e. IDX in 2010 or later and GPD data available in the year before). Numbers are counts (N), percentage and 95% CI.

|  | **Linked study population (i.e. IDX in 2010 or later and GPD data available in the year before the IDX)** | | | |
| --- | --- | --- | --- | --- |
|  | N | **Cases** | N | **Controls** |
|  | % | [95% CI] | % | [95% CI] |
| **Total** | **1721** |  | **8605** |  |
| **Males/Females** | | | | |
| Females | 359 |  | 1795 |  |
|  | 20.9 | [19.1, 22.9] | 20.9 | [20.1, 21.8] |
| Males | 1362 |  | 6810 |  |
|  | 79.2 | [77.2, 81.0] | 79.2 | [78.3, 80.0] |
| **Age group** | | | | |
| Children & young people: [10, 24] y.o. | 164 |  | 820 |  |
|  | 9.6 | [8.3, 11.1] | 9.6 | [9.0, 10.2] |
| Adults: [25, 64] y.o. | 1253 |  | 6272 |  |
|  | 72.9 | [70.7, 74.9] | 72.9 | [72.0, 73.9] |
| Older population: 64+ y.o. | 304 |  | 1513 |  |
|  | 17.7 | [16.0, 19.6] | 17.6 | [16.8, 18.5] |
| **WIMD deprivation** | | | | |
| Least deprived - 1 | 247 |  | 1799 |  |
|  | 14.4 | [12.8, 16.1] | 21 | [20.1, 21.8] |
| 2 | 282 |  | 1552 |  |
|  | 16.4 | [14.8, 18.3] | 18.1 | [17.3, 18.9] |
| 3 | 348 |  | 1797 |  |
|  | 20.3 | [18.4, 22.2] | 20.9 | [20.1, 21.8] |
| 4 | 360 |  | 1699 |  |
|  | 21 | [19.1, 23.0] | 19.8 | [19.0, 20.6] |
| Most deprived - 5 | 414 |  | 1749 |  |
|  | 24.1 | [22.1, 26.2] | 20.4 | [19.5, 21.2] |
| **Area morphology** | | | | |
| Urban | 1140 |  | 5959 |  |
|  | 66.3 | [64.0, 68.5] | 69.3 | [68.3, 70.3] |
| Rural | 511 |  | 2637 |  |
|  | 29.7 | [27.6, 31.9] | 30.7 | [29.7, 31.7] |

**Supplementary Table 5:** Type of GP contacts in the year before IDX for females and males with GPD data available in this period. Numbers are counts (N), percentages (%), 95% CI and odds ratios (OR) adjusted for deprivation (p<0.001 except otherwise specified). Specified models did not converge (DNC).

|  | **Cases** | | | |  | **Controls** | | | |  | **Odds Ratios** | | | |
| --- | --- | --- | --- | --- | --- | --- | --- | --- | --- | --- | --- | --- | --- | --- |
|  | N | **Females** | N | **Males** |  | N | **Females** | N | **Males** |  | **Females** | | **Males** | |
|  | % | [95% CI] | % | [95% CI] |  | % | [95% CI] | % | [95% CI] |  | OR | 95% CI | OR | 95% CI |
| **Total** | **779** |  | **2725** |  |  | **3895** |  | **13625** |  |  |  |  |  |  |
| Any contact | 755 |  | 2374 |  |  | 3583 |  | 10359 |  |  | 2.9 | [2.3, 3.6] | 2.2 | [2.0, 2.3] |
|  |  | [95.5, 98.0] |  | [85.9, 88.4] |  | 92 | [91.1, 92.9] | 76.1 | [75.4, 76.8] |  |  | |  | |
| Recorded mental health specialty contact | 117 |  | 242 |  |  | 48 |  | 113 |  |  | 14.4 | [10.4, 19.8] | 11.7 | [9.5, 14.3] |
|  | 15.1 | [12.7, 17.7] | 8.9 | [7.9, 10.1] |  | 1.3 | [1.0, 1.7] | 0.9 | [0.7, 1.0] |  |  | |  | |
| **Diagnoses** | | | | | | | | | | | | | | |
| Any diagnosis | 748 |  | 2322 |  |  | 3533 |  | 10027 |  |  | 2.6 | [2.1, 3.1] | 2.1 | [2.0, 2.3] |
|  | 96.1 | [94.5, 97.2] | 85.3 | [83.9, 86.5] |  | 90.8 | [89.8, 91.6] | 73.6 | [72.9, 74.4] |  |  | |  | |
| Non-mental health | 748 |  | 2307 |  |  | 3532 |  | 10010 |  |  | 2.6 | [2.1, 3.1] | 2 | [1.9, 2.2] |
|  | 96.1 | [94.5, 97.2] | 84.7 | [83.3, 86.0] |  | 90.7 | [89.8, 91.6] | 73.5 | [72.8, 74.3] |  |  | |  | |
| Mental health | 266 |  | 741 |  |  | 278 |  | 699 |  |  | 6.6 | [5.6, 7.7] | 7 | [6.4, 7.7] |
|  | 34.2 | [31.0, 37.6] | 27.2 | [25.6, 28.9] |  | 7.2 | [6.4, 8.0] | 5.2 | [4.8, 5.6] |  |  | |  | |
| Common mental disorder | 214 |  | 553 |  |  | 225 |  | 437 |  |  | 6.1 | [5.1, 7.2] | 7.8 | [7.0, 8.7] |
|  | 27.5 | [24.5, 30.8] | 20.3 | [18.9, 21.9] |  | 5.8 | [5.1, 6.6] | 3.3 | [3.0, 3.6] |  |  | |  | |
| Injury & poisoning | 53 |  | 175 |  |  | 164 |  | 588 |  |  | 1.7 | [1.4, 2.0] | 1.5 | [1.4, 1.7] |
|  | 6.9 | [5.3, 8.8] | 6.5 | [5.6, 7.5] |  | 4.3 | [3.7, 4.9] | 4.4 | [4.0, 4.7] |  |  | |  | |
| Accidents | 34 |  | 49 |  |  | 69 |  | 180 |  |  | 2.6 | [1.9, 3.5] | 1.3 | [1.1, 1.6] |
|  | 4.4 | [3.2, 6.1] | 1.8 | [1.4, 2.4] |  | 1.8 | [1.5, 2.3] | 1.4 | [1.2, 1.6] |  |  | | p=0.006 | |
| Accidental hanging & poisoning | <5 |  | <5 |  |  | 0 |  | <5 |  |  |  |  | 2.2 | [0.4, 11.2] |
|  | 0.7 | [0.3, 1.5] | 0.2 | [0.1, 0.5] |  | 0 | [0.0, 0.1] | 0.1 | [0.1, 0.1] |  |  | | p=0.356 | |
| Self-harm | 83 |  | 169 |  |  | 9 |  | 29 |  |  | 44.6 | [23.0, 86.4] | 29.4 | [20.1, 42.8] |
|  | 10.7 | [8.7, 13.1] | 6.3 | [5.4, 7.2] |  | 0.3 | [0.2, 0.5] | 0.3 | [0.2, 0.4] |  |  | |  | |
| Alcohol misuse | 48 |  | 146 |  |  | <5 |  | 94 |  |  | 225.7 | [31.6, 1609.1] | 7.7 | [6.2, 9.7] |
|  | 6.2 | [4.7, 8.1] | 5.4 | [4.6, 6.3] |  | 0.2 | [0.1, 0.4] | 0.7 | [0.6, 0.9] |  |  | |  | |
| Drugs misuse | 12 |  | 75 |  |  | 7 |  | 62 |  |  | 7.9 | [3.6, 17.3] | 5.9 | [4.5, 7.7] |
|  | 1.6 | [0.9, 2.7] | 2.8 | [2.3, 3.5] |  | 0.2 | [0.1, 0.4] | 0.5 | [0.4, 0.6] |  |  | |  | |
| **Prescriptions** | | | | | | | | | | | | | | |
| Any prescription | 755 |  | 2359 |  |  | 3564 |  | 10212 |  |  | 3 | [2.4, 3.8] | 2.2 | [2.0, 2.3] |
|  | 97 | [95.5, 98.0] | 86.6 | [85.3, 87.8] |  | 91.6 | [90.6, 92.4] | 75 | [74.3, 75.7] |  |  | |  | |
| Opiates | 286 |  | 632 |  |  | 888 |  | 1945 |  |  | 1.9 | [1.7, 2.1] | 1.8 | [1.7, 1.9] |
|  | 36.8 | [33.5, 40.2] | 23.2 | [21.7, 24.9] |  | 22.8 | [21.6, 24.2] | 14.3 | [13.7, 14.9] |  |  | |  | |
| Psychotropics | 585 |  | 1386 |  |  | 926 |  | 1648 |  |  | 9.5 | [8.3, 10.8] | 7.7 | [7.1, 8.3] |
|  | 75.1 | [72.0, 78.1] | 50.9 | [49.0, 52.8] |  | 23.8 | [22.5, 25.2] | 12.1 | [11.6, 12.7] |  |  | |  | |

**Supplementary Table 6:** Type of hospital admissions in the year before IDX for females and males. Numbers are counts (N), percentage and 95% CI and odds ratios (OR) adjusted for deprivation (p<0.001 except otherwise specified). Specified models did not converge (DNC).

|  | **Cases** | | | |  | **Controls** | | | |  | **Odds Ratios** | | | |
| --- | --- | --- | --- | --- | --- | --- | --- | --- | --- | --- | --- | --- | --- | --- |
|  | N | **Females** | N | **Males** |  | N | **Females** | N | **Males** |  | **Females** | | **Males** | |
|  | % | [95% CI] | % | [95% CI] |  | % | [95% CI] | % | [95% CI] |  | OR | 95% CI | OR | 95% CI |
| **Total** | **1131** |  | **3999** |  |  | **5655** |  | **19995** |  |  |  |  |  |  |
| Any contact | 518 |  | 1244 |  |  | 968 |  | 2438 |  |  | 4 | [3.6, 4.4] | 3.3 | [3.1, 3.5] |
|  | 45.9 | [43.0, 48.8] | 31.2 | [29.7, 32.6] |  | 17.2 | [16.2, 18.2] | 12.2 | [11.8, 12.7] |  |  | |  | |
| Mental health specialty | 168 |  | 330 |  |  | 15 |  | 34 |  |  | 63.2 | [36.5, 109.3] | 49.4 | [35.0, 69.7] |
|  | 14.9 | [13.0, 17.1] | 8.3 | [7.5, 9.2] |  | 0.3 | [0.2, 0.5] | 0.2 | [0.2, 0.3] |  |  | |  | |
| **Emergency admissions by diagnosis** | | | | | | | | | | | | | | |
| Any | 426 |  | 1035 |  |  | 378 |  | 1113 |  |  | 8.1 | [7.1, 9.3] | 5.9 | [5.5, 6.4] |
|  | 37.7 | [34.9, 40.6] | 25.9 | [24.6, 27.3] |  | 6.7 | [6.1, 7.4] | 5.6 | [5.3, 5.9] |  |  | |  | |
| Mental health | 293 |  | 605 |  |  | 80 |  | 202 |  |  | 22.3 | [17.3, 28.7] | 17.2 | [14.7, 20.1] |
|  | 26 | [23.5, 28.6] | 15.2 | [14.1, 16.3] |  | 1.5 | [1.2, 1.8] | 1.1 | [0.9, 1.2] |  |  | |  | |
| Common mental disorder | 181 |  | 316 |  |  | 32 |  | 58 |  |  | 33.8 | [22.9, 50.0] | 28.4 | [21.7, 37.1] |
|  | 16.1 | [14.0, 18.3] | 8 | [7.2, 8.8] |  | 0.6 | [0.5, 0.8] | 0.3 | [0.3, 0.4] |  |  | |  | |
| Injury & poisoning | 221 |  | 488 |  |  | 61 |  | 254 |  |  | 19.8 | [15.1, 26.0] | 10.4 | [9.1, 12.0] |
|  | 19.6 | [17.4, 22.0] | 12.3 | [11.3, 13.3] |  | 1.1 | [0.9, 1.4] | 1.3 | [1.2, 1.5] |  |  | |  | |
| Accidents | 62 |  | 181 |  |  | 44 |  | 186 |  |  | 6.9 | [5.0, 9.5] | 4.8 | [4.1, 5.7] |
|  | 5.5 | [4.3, 7.0] | 4.6 | [4.0, 5.3] |  | 0.8 | [0.6, 1.1] | 1 | [0.9, 1.1] |  |  | |  | |
| Accidental hanging & poisoning | 28 |  | 54 |  |  | <5 |  | 8 |  |  | 128.7 | [18.0, 920.4] | 31.7 | [15.7, 64.1] |
|  | 2.5 | [1.8, 3.6] | 1.4 | [1.1, 1.8] |  | 0.1 | [0.1, 0.3] | 0.1 | [0.1, 0.1] |  |  | |  | |
| Self-harm | 169 |  | 302 |  |  | 8 |  | 18 |  |  | 103.2 | [51.4, 207.0] | 90.9 | [55.5, 148.7] |
|  | 15 | [13.0, 17.2] | 7.6 | [6.8, 8.5] |  | 0.2 | [0.1, 0.3] | 0.1 | [0.1, 0.2] |  |  | |  | |
| Alcohol misuse | 77 |  | 201 |  |  | 10 |  | 65 |  |  | 45 | [22.4, 90.8] | 16.6 | [12.7, 21.7] |
|  | 6.9 | [5.5, 8.5] | 5.1 | [4.4, 5.8] |  | 0.2 | [0.1, 0.4] | 0.4 | [0.3, 0.5] |  |  | |  | |
| Drugs misuse | 20 |  | 77 |  |  | <5 |  | 24 |  |  | 45.4 | [11.2, 184.2] | 14.9 | [9.9, 22.6] |
|  | 1.8 | [1.2, 2.8] | 2 | [1.6, 2.4] |  | 0.1 | [0.1, 0.3] | 0.2 | [0.1, 0.2] |  |  | |  | |
| **Elective admissions by diagnosis** | | | | | | | | | | | | | | |
| Any | 193 |  | 415 |  |  | 584 |  | 1603 |  |  | 1.8 | [1.6, 2.0] | 1.4 | [1.3, 1.5] |
|  | 17.1 | [15.0, 19.4] | 10.4 | [9.5, 11.4] |  | 10.4 | [9.6, 11.2] | 8.1 | [7.7, 8.5] |  |  | |  | |
| Mental health | 53 |  | 81 |  |  | 40 |  | 70 |  |  | 6.6 | [4.7, 9.2] | 5.9 | [4.6, 7.6] |
|  | 4.7 | [3.7, 6.1] | 2.1 | [1.7, 2.6] |  | 0.8 | [0.6, 1.0] | 0.4 | [0.3, 0.5] |  |  | |  | |
| Common mental disorder | 31 |  | 25 |  |  | 13 |  | 18 |  |  | 11.9 | [6.7, 20.9] | 7.2 | [4.4, 11.9] |
|  | 2.8 | [2.0, 3.9] | 0.7 | [0.5, 1.0] |  | 0.3 | [0.2, 0.4] | 0.1 | [0.1, 0.2] |  |  | |  | |
| Alcohol misuse | 6 |  | 18 |  |  | <5 |  | 9 |  |  | 23.9 | [3.3, 176.0] | 9.9 | [5.0, 19.7] |
|  | 0.6 | [0.3, 1.2] | 0.5 | [0.3, 0.8] |  | 0.1 | [0.1, 0.3] | 0.1 | [0.1, 0.1] |  | p=0.002 | |  | |
| Drugs misuse | <5 |  | 7 |  |  | 0 |  | <5 |  |  | DNC |  | 9.9 | [3.0, 32.2] |
|  | 0.5 | [0.2, 1.1] | 0.2 | [0.1, 0.4] |  | 0 | [-0.0, 0.1] | 0.1 | [0.1, 0.1] |  |  | |  | |

**Supplementary Table 7:** Type of health setting contacted before the IDX for the fully linked study population (i.e. those with IDX in 2010 or later and GPD data available in the year before the IDX). Numbers are counts (N), percentages and 95% CI and odds ratios (OR) adjusted for deprivation (p<0.001 except otherwise specified).

|  | **Cases** | | | |  | **Controls** | | | |  | **Odds Ratios** | | | |
| --- | --- | --- | --- | --- | --- | --- | --- | --- | --- | --- | --- | --- | --- | --- |
|  | N | **Females** | N | **Males** |  | N | **Females** | N | **Males** |  | **Females** | | **Males** | |
|  | % | [95% CI] | % | [95% CI] |  | % | [95% CI] | % | [95% CI] |  | OR | 95% CI | OR | 95% CI |
| **Total** | **359** |  | **1362** |  |  | **1795** |  | **6810** |  |  |  |  |  |  |
| **Any healthcare setting** | | | | | | | | | | | | | | |
| 1 week | 202 |  | 460 |  |  | 461 |  | 1202 |  |  | 3.8 | [3.2, 4.5] | 2.5 | [2.3, 2.7] |
|  | 56.3 | [51.1, 61.4] | 33.8 | [31.4, 36.4] |  | 25.7 | [23.8, 27.8] | 17.7 | [16.8, 18.6] |  |  | |  | |
| 1 month | 337 |  | 1006 |  |  | 1322 |  | 3613 |  |  | 6 | [4.6, 7.7] | 2.7 | [2.5, 2.9] |
|  | 93.9 | [90.9, 96.0] | 73.9 | [71.5, 76.2] |  | 73.7 | [71.6, 75.7] | 53.1 | [51.9, 54.3] |  |  | |  | |
| 1 year | 356 |  | 1249 |  |  | 1705 |  | 5362 |  |  | 6.2 | [3.5, 10.9] | 3 | [2.7, 3.4] |
|  | 99.2 | [97.6, 99.8] | 91.8 | [90.2, 93.1] |  | 95 | [93.9, 96.0] | 78.8 | [77.8, 79.7] |  |  | |  | |
| **GP** | | | | | | | | | | | | | | |
| 1 week | 186 |  | 416 |  |  | 434 |  | 1109 |  |  | 3.5 | [3.0, 4.1] | 2.3 | [2.1, 2.6] |
|  | 51.9 | [46.7, 57.0] | 30.6 | [28.2, 33.1] |  | 24.2 | [22.3, 26.3] | 16.3 | [15.5, 17.2] |  |  | |  | |
| 1 month | 327 |  | 966 |  |  | 1300 |  | 3508 |  |  | 4.3 | [3.5, 5.4] | 2.5 | [2.3, 2.7] |
|  | 91.1 | [87.7, 93.7] | 71 | [68.5, 73.3] |  | 72.5 | [70.4, 74.5] | 51.6 | [50.4, 52.7] |  |  | |  | |
| 1 year | 352 |  | 1209 |  |  | 1692 |  | 5243 |  |  | 3.2 | [2.1, 4.7] | 2.4 | [2.2, 2.6] |
|  | 98.1 | [96.1, 99.1] | 88.8 | [87.0, 90.4] |  | 94.3 | [93.1, 95.3] | 77 | [76.0, 78.0] |  |  | |  | |
| **ED** | | | | | | | | | | | | | | |
| 1 week | 28 |  | 49 |  |  | 8 |  | 34 |  |  | 20 | [9.3, 42.7] | 7.9 | [5.4, 11.4] |
|  | 7.8 | [5.5, 11.1] | 3.6 | [2.8, 4.8] |  | 0.5 | [0.3, 0.9] | 0.5 | [0.4, 0.7] |  |  | |  | |
| 1 month | 87 |  | 198 |  |  | 70 |  | 242 |  |  | 8.4 | [6.2, 11.2] | 4.8 | [4.1, 5.6] |
|  | 24.3 | [20.1, 29.0] | 14.6 | [12.8, 16.6] |  | 3.9 | [3.1, 4.9] | 3.6 | [3.2, 4.1] |  |  | |  | |
| 1 year | 182 |  | 530 |  |  | 296 |  | 1130 |  |  | 5.4 | [4.5, 6.5] | 3.2 | [2.9, 3.4] |
|  | 50.7 | [45.6, 55.9] | 39 | [36.4, 41.6] |  | 16.5 | [14.9, 18.3] | 16.6 | [15.8, 17.5] |  |  | |  | |
| **Hospital inpatients** | | | | | | | | | | | | | | |
| 1 week | 20 |  | 48 |  |  | 16 |  | 50 |  |  | 7.3 | [4.2, 12.6] | 5.4 | [3.9, 7.4] |
|  | 5.6 | [3.7, 8.5] | 3.6 | [2.7, 4.7] |  | 0.9 | [0.6, 1.5] | 0.8 | [0.6, 1.0] |  |  | |  | |
| 1 month | 70 |  | 153 |  |  | 85 |  | 218 |  |  | 4.7 | [3.7, 6.1] | 4 | [3.4, 4.7] |
|  | 19.5 | [15.8, 24.0] | 11.3 | [9.7, 13.1] |  | 4.8 | [3.9, 5.9] | 3.3 | [2.9, 3.7] |  |  | |  | |
| 1 year | 165 |  | 404 |  |  | 329 |  | 854 |  |  | 3.5 | [3.0, 4.1] | 3 | [2.7, 3.3] |
|  | 46 | [40.9, 51.2] | 29.7 | [27.3, 32.2] |  | 18.4 | [16.7, 20.2] | 12.6 | [11.8, 13.4] |  |  | |  | |
| **Hospital outpatients** | | | | | | | | | | | | | | |
| 1 week | 19 |  | 30 |  |  | 39 |  | 100 |  |  | 2.6 | [1.8, 3.8] | 1.5 | [1.2, 2.0] |
|  | 5.3 | [3.5, 8.2] | 2.3 | [1.6, 3.2] |  | 2.2 | [1.6, 3.0] | 1.5 | [1.3, 1.8] |  |  | | p=0.001 | |
| 1 month | 97 |  | 191 |  |  | 256 |  | 614 |  |  | 2.2 | [1.8, 2.6] | 1.7 | [1.5, 1.9] |
|  | 27.1 | [22.7, 31.9] | 14.1 | [12.3, 16.0] |  | 14.3 | [12.8, 16.0] | 9.1 | [8.4, 9.8] |  |  | |  | |
| 1 year | 205 |  | 509 |  |  | 701 |  | 1808 |  |  | 2.1 | [1.8, 2.4] | 1.7 | [1.6, 1.9] |
|  | 57.2 | [52.0, 62.2] | 37.4 | [34.9, 40.0] |  | 39.1 | [36.9, 41.4] | 26.6 | [25.6, 27.7] |  |  | |  | |

**Supplementary Table 8**. Demographic information of the sensitivity study population, including only those with a coroner’s conclusion of ‘suicide’. Full sensitivity study population and those with fully linked data (i.e. IDX in 2010 or later and GPD data available in the year before). Numbers are counts (N), percentage and 95% CI.

|  | **Sensitivity study population**  **(i.e. coroner’s conclusion of ‘suicide’)** | | | |  | **Linked sensitivity study population (i.e. coroner’s conclusion of ‘suicide’, IDX in 2010 or later and WGP data available in the year before the IDX)** | | | |
| --- | --- | --- | --- | --- | --- | --- | --- | --- | --- |
|  | N | **Cases** | N | **Controls** |  | N | **Cases** | N | **Controls** |
|  | % | [95% CI] | % | [95% CI] |  | % | [95% CI] | % | [95% CI] |
| **Total** | **4091** |  | **20455** |  |  | **1362** |  | **6810** |  |
| **Males/Females** | | | | | | | | | |
| Females | 804 |  | 4020 |  |  | 246 |  | 1230 |  |
|  | 19.7 | [18.5, 20.9] | 19.7 | [19.2, 20.3] |  | 18.1 | [16.2, 20.2] | 18.1 | [17.2, 19.0] |
| Males | 3287 |  | 16435 |  |  | 1116 |  | 5580 |  |
|  | 80.4 | [79.2, 81.6] | 80.4 | [79.8, 80.9] |  | 82 | [79.9, 83.9] | 82 | [81.1, 82.9] |
| **Age group** | | | | | | | | | |
| Children & young people: [10, 24] y.o. | 431 |  | 2156 |  |  | 125 |  | 625 |  |
|  | 10.6 | [9.7, 11.6] | 10.6 | [10.2, 11.0] |  | 9.2 | [7.8, 10.9] | 9.2 | [8.6, 9.9] |
| Adults: [25, 64] y.o. | 3020 |  | 15107 |  |  | 1001 |  | 5012 |  |
|  | 73.9 | [72.5, 75.2] | 73.9 | [73.3, 74.5] |  | 73.5 | [71.1, 75.8] | 73.6 | [72.6, 74.7] |
| Older population: 64+ y.o. | 640 |  | 3192 |  |  | 236 |  | 1173 |  |
|  | 15.7 | [14.6, 16.8] | 15.7 | [15.2, 16.2] |  | 17.4 | [15.5, 19.5] | 17.3 | [16.4, 18.2] |
| **WIMD deprivation** | | | | | | | | | |
| Least deprived - 1 | 558 |  | 4263 |  |  | 192 |  | 1386 |  |
|  | 13.7 | [12.7, 14.8] | 20.9 | [20.3, 21.5] |  | 14.1 | [12.4, 16.1] | 20.4 | [19.5, 21.4] |
| 2 | 689 |  | 3865 |  |  | 227 |  | 1220 |  |
|  | 16.9 | [15.8, 18.1] | 18.9 | [18.4, 19.5] |  | 16.7 | [14.8, 18.8] | 18 | [17.1, 18.9] |
| 3 | 760 |  | 4209 |  |  | 272 |  | 1432 |  |
|  | 18.6 | [17.5, 19.8] | 20.6 | [20.1, 21.2] |  | 20 | [18.0, 22.2] | 21.1 | [20.1, 22.1] |
| 4 | 862 |  | 4066 |  |  | 297 |  | 1361 |  |
|  | 21.1 | [19.9, 22.4] | 19.9 | [19.4, 20.5] |  | 21.9 | [19.7, 24.1] | 20 | [19.1, 21.0] |
| Most deprived - 5 | 987 |  | 4029 |  |  | 321 |  | 1404 |  |
|  | 24.2 | [22.9, 25.5] | 19.7 | [19.2, 20.3] |  | 23.6 | [21.4, 25.9] | 20.7 | [19.7, 21.6] |
| **Area morphology** | | | | | | | | | |
| Urban | 2574 |  | 13918 |  |  | 894 |  | 4707 |  |
|  | 63 | [61.5, 64.4] | 68.1 | [67.4, 68.7] |  | 65.7 | [63.1, 68.2] | 69.2 | [68.1, 70.3] |
| Rural | 670 |  | 3325 |  |  | 214 |  | 1103 |  |
|  | 16.4 | [15.3, 17.6] | 16.3 | [15.8, 16.8] |  | 15.8 | [13.9, 17.8] | 16.2 | [15.4, 17.1] |

**Supplementary Table 9:** Type of health setting contacted before the IDX for the fully linked sensitivity study population (i.e. those with coroner’s conclusion of ‘suicide’, IDX in 2010 or later and GPD data available in the year before the IDX). Numbers are counts (N), percentages and 95% CI and odds ratios (OR) adjusted for deprivation (p<0.001 except otherwise specified).

|  | N | **Cases** |  | N | **Controls** |  | **OR** | **95% CI** |
| --- | --- | --- | --- | --- | --- | --- | --- | --- |
|  | % | [95% CI] |  | % | [95% CI] |  |  |  |
| **Total** | **1362** |  |  | **6810** |  |  |  |  |
| **Any healthcare setting** | | | | | | | | |
| 1 week | 520 |  |  | 1305 |  |  | 2.7 | [2.5, 3.0] |
|  | 38.2 | [35.7, 40.8] |  | 19.2 | [18.3, 20.2] |  |  | |
| 1 month | 1045 |  |  | 3882 |  |  | 2.8 | [2.6, 3.0] |
|  | 76.8 | [74.5, 78.9] |  | 57.1 | [55.9, 58.2] |  |  | |
| 1 year | 1268 |  |  | 5565 |  |  | 3.1 | [2.8, 3.5] |
|  | 93.1 | [91.7, 94.4] |  | 81.8 | [80.8, 82.7] |  |  | |
| **GP** | | | | | | | | |
| 1 week | 474 |  |  | 1208 |  |  | 2.6 | [2.4, 2.8] |
|  | 34.9 | [32.4, 37.4] |  | 17.8 | [16.9, 18.7] |  |  | |
| 1 month | 1008 |  |  | 3777 |  |  | 2.6 | [2.4, 2.8] |
|  | 74.1 | [71.7, 76.3] |  | 55.5 | [54.3, 56.7] |  |  | |
| 1 year | 1230 |  |  | 5457 |  |  | 2.4 | [2.1, 2.6] |
|  | 90.4 | [88.7, 91.8] |  | 80.2 | [79.2, 81.1] |  |  | |
| **ED** | | | | | | | | |
| 1 week | 54 |  |  | 37 |  |  | 8.1 | [5.7, 11.6] |
|  | 4 | [3.1, 5.2] |  | 0.6 | [0.4, 0.8] |  |  | |
| 1 month | 218 |  |  | 238 |  |  | 5.6 | [4.8, 6.5] |
|  | 16.1 | [14.2, 18.1] |  | 3.5 | [3.1, 4.0] |  |  | |
| 1 year | 544 |  |  | 1121 |  |  | 3.4 | [3.1, 3.7] |
|  | 40 | [37.4, 42.6] |  | 16.5 | [15.6, 17.4] |  |  | |
| **Hospital inpatients** | | | | | | | | |
| 1 week | 57 |  |  | 52 |  |  | 5.9 | [4.4, 8.0] |
|  | 4.2 | [3.3, 5.4] |  | 0.8 | [0.6, 1.0] |  |  | |
| 1 month | 178 |  |  | 239 |  |  | 4.3 | [3.7, 5.0] |
|  | 13.1 | [11.4, 15.0] |  | 3.6 | [3.1, 4.0] |  |  | |
| 1 year | 453 |  |  | 912 |  |  | 3.2 | [3.0, 3.6] |
|  | 33.3 | [30.9, 35.9] |  | 13.4 | [12.7, 14.3] |  |  | |
| **Hospital outpatients** | | | | | | | | |
| 1 week | 43 |  |  | 113 |  |  | 2 | [1.6, 2.5] |
|  | 3.2 | [2.4, 4.3] |  | 1.7 | [1.4, 2.0] |  |  | |
| 1 month | 224 |  |  | 676 |  |  | 1.8 | [1.6, 2.0] |
|  | 16.5 | [14.6, 18.6] |  | 10 | [9.3, 10.7] |  |  | |
| 1 year | 558 |  |  | 1970 |  |  | 1.8 | [1.7, 1.9] |
|  | 41 | [38.4, 43.7] |  | 29 | [27.9, 30.1] |  |  | |

**[attached separately]**

**Supplementary Figure 1:** Flow diagram showing the size of the study population at each stage and the coverage of different datasets: Welsh Demographic Service (WDS), Office for National Statistics (ONS) - Deaths, General Practice Database (GPD), Emergency Department Data Set (EDDS), Patient Episode Database for Wales (PEDW), Outpatient Dataset (OPD).

**[attached separately]**

**Supplementary Figure 2:** Rate of cases and controls with mental health (left) and self-harm (right) contacts in any health care setting in the [1, 365] days before the IDX (top) and in the week starting [7, 365] days before the IDX (bottom). p-values correspond to conditional logistic regression adjusted for deprivation.
