# Supplementary material for: Contacts with primary and secondary healthcare prior to suicide: case–control whole-population-based study using person-level linked routine data in Wales, UK, 2000–2017
Source: Br J Psychiatry. 2020 Aug 3;217(6):717–24. doi: 10.1192/bjp.2020.137 (PMC7705668; doi:10.1192/bjp.2020.137)
Supplement: Supplementary file 1 [file S0007125020001373sup001.zip › S0007125020001373sup003.pdf]

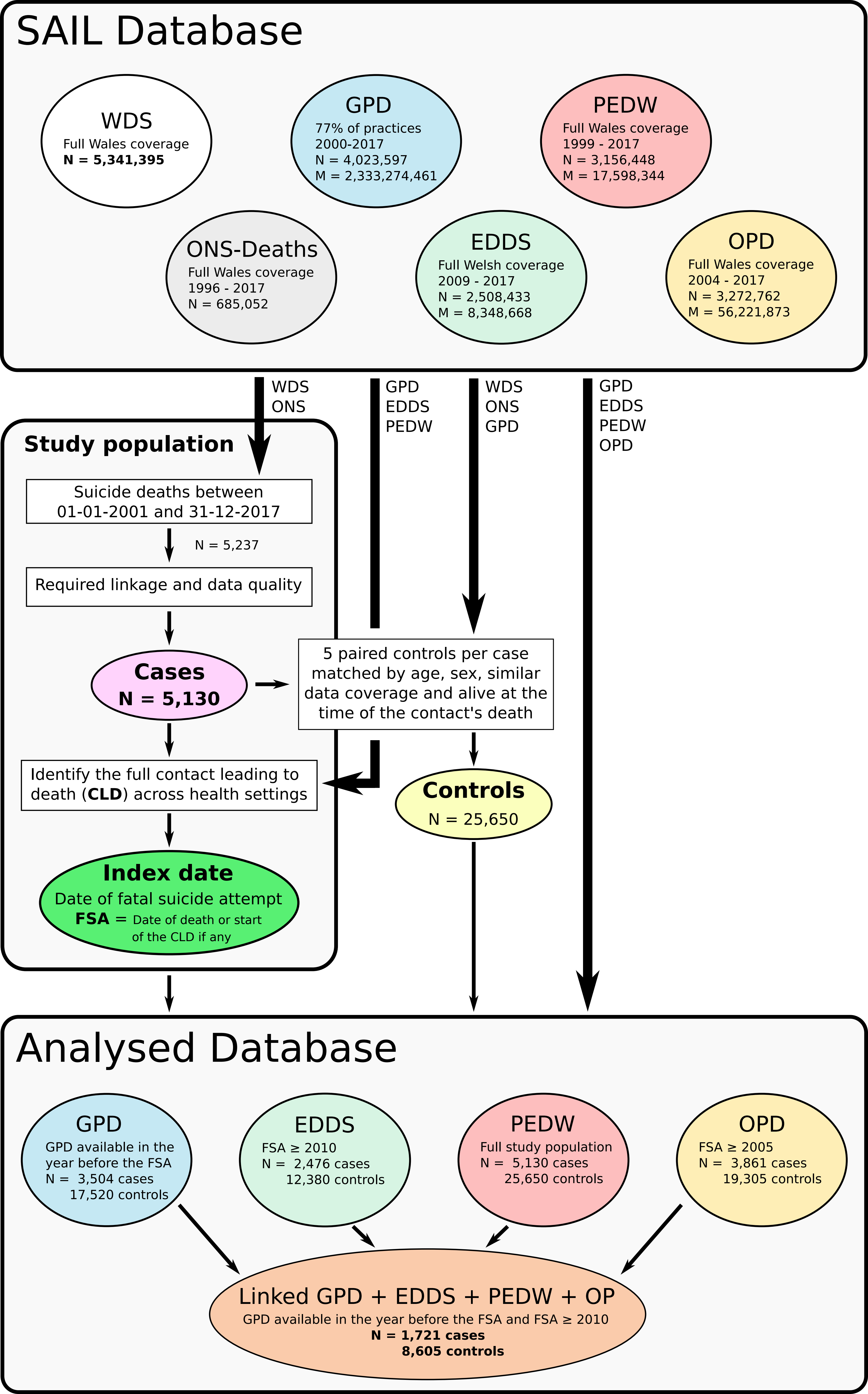

SAIL Database
WDS
Full Wales coverage
N = 5,341,395
GPD
77% of practices
2000-2017
N = 4,023,597
M = 2,333,274,461
PEDW
Full Wales coverage
1999 - 2017
N = 3,156,448
M = 17,598,344
ONS-Deaths
Full Wales coverage
1996 - 2017
N = 685,052
EDDS
Full Welsh coverage
2009 - 2017
N = 2,508,433
M = 8,348,668
OPD
Full Wales coverage
2004 - 2017
N = 3,272,762
M = 56,221,873
WDS
ONS
GPD
EDDS
PEDW
WDS
ONS
GPD
GPD
EDDS
PEDW
OPD
Study population
Suicide deaths between 01-01-2001 and 31-12-2017
N = 5,237
Required linkage and data quality
Cases
N = 5,130
5 paired controls per case matched by age, sex, similar data coverage and alive at the time of the contact's death
Identify the full contact leading to death (CLD) across health settings
Controls
N = 25,650
Index date
Date of fatal suicide attempt
FSA = Date of death or start of the CLD if any
Analysed Database
GPD
GPD available in the year before the FSA
N = 3,504 cases
17,520 controls
EDDS
FSA ≥ 2010
N = 2,476 cases
12,380 controls
PEDW
Full study population
N = 5,130 cases
25,650 controls
OPD
FSA ≥ 2005
N = 3,861 cases
19,305 controls
Linked GPD + EDDS + PEDW + OP
GPD available in the year before the FSA and FSA ≥ 2010
N = 1,721 cases
8,605 controls
