# Supplementary material for: Contacts with primary and secondary healthcare prior to suicide: case–control whole-population-based study using person-level linked routine data in Wales, UK, 2000–2017
Source: Br J Psychiatry. 2020 Aug 3;217(6):717–24. doi: 10.1192/bjp.2020.137 (PMC7705668; doi:10.1192/bjp.2020.137)
Supplement: Supplementary file 1 [file S0007125020001373sup001.zip › S0007125020001373sup004.pdf]

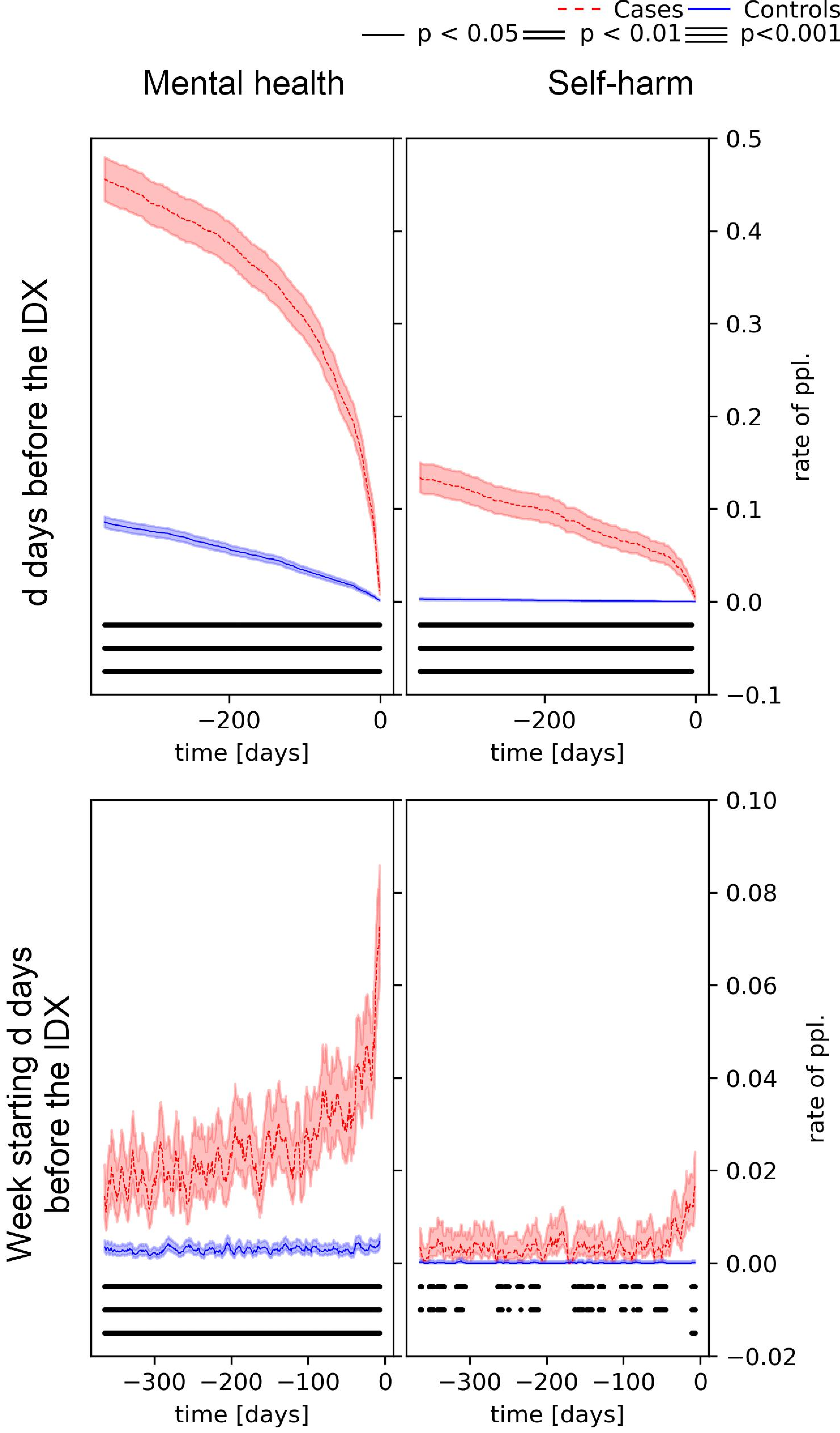

Cases
Controls
p < 0.05
p < 0.01
p<0.001
Mental health
Self-harm
d days before the IDX
Week starting d days before the IDX
rate of ppl.
rate of ppl.
time [days]
time [days]
time [days]
time [days]
0.5
0.4
0.3
0.2
0.1
0.0
−0.1
0.10
0.08
0.06
0.04
0.02
0.00
−0.02
−200
0
−300
−200
−100
0
